# Virtual Lobule Models Are the Key for Multiscale Biomechanical and Pharmacological Modeling for the Liver

**DOI:** 10.3389/fphys.2020.01061

**Published:** 2020-09-02

**Authors:** Harvey Ho, En Zhang

**Affiliations:** ^1^Bioengineering Institute, The University of Auckland, Auckland, New Zealand; ^2^Chongqing Institute for Food and Drug Control, Chongqing City, China

**Keywords:** liver lobule, multiscale modeling, hepatic circulation, pharmacokinetics, drug

## Introduction

**Figure 1:**
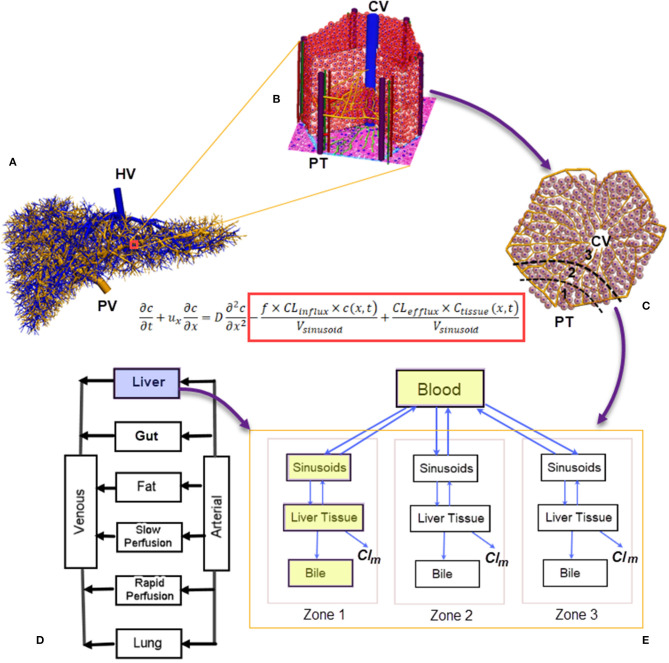
A numerical scheme for multiscale biomechanical and pharmacological modeling for the liver: **(A)** Vasculatures generated from the CCO algorithm ranging from root vessels to portal triads at the peripherals of liver lobules (Barléon et al., 2018); **(B)** a 3D representation of the liver lobule; **(C)** a 2D cross-section of the liver lobule, highlighting the three metabolic zones; **(D)** a PBPK model where the liver is modeled as a “well-stirred” organ. Liver-specific PK can be modeled through **(E)** a compartmental model for drug clearance in the liver incorporating the blood, sinusoids, tissue, and bile compartments (shown in yellow blocks). Spatial heterogeneity in metabolism can be simulated by arranging the compartments in a series with different metabolism and clearance parameters (Meyer et al., 2017). In the drug-transport equation, the influx/efflux of drug molecules across the sinusoidal wall are included inside the red block (Franiatte et al., 2019). PT, portal triad; CV, central vein; *Cl*_*m*_, drug clearance through metabolism; CCO, constructive constrained optimisation; PBPK, pharmacokinetics based pharmacokinetic modeling.

The liver has a unique dual blood-supply system of hepatic arterial (HA) and portal venous (PV) vasculatures, which are drained by a hepatic venous (HV) tree (Figure 1A). The blood reaches the peripheral portal triads (PT) of ~10^5^ lobules, the functioning units of the liver (Ohno et al., 2008). With a diameter of 1.0–1.3 mm (Ricken et al., 2015), each lobule consists of ~10^6^ hepatocytes and 1,000 sinusoids (Wambaugh and Shah, 2010; Fu et al., 2018), which merge into central veins (CV) (Ho et al., 2013a; Sluka et al., 2016), as shown in Figure 1B. The perfusion and active transport of drug molecules between the blood and hepatocytes occur at sinusoids, which exhibit spatial heterogeneity in transporters. Hepatocytes in a lobule are conventionally grouped into three metabolic zones, namely Zones 1, 2, and 3 from the PT to the CV (Figure 1C) (Jungermann, 1995). Such a zonal differentiation is crucial in spatially heterogeneous liver diseases. For example, an overdose of the painkiller acetaminophen may lead to hepatotoxicity and necrosis of hepatocytes, which mostly occur at Zone 3 (Means and Ho, 2019). Pediatric patients with non-alcoholic fatty liver disease may show a higher prevalence of Zone 1 steatosis and periportal fibrosis as compared with adult populations (Kleiner and Brunt, 2012).

*In silico* liver models are increasingly used to simulate hemodynamic and pharmacological phenomena (Ricken et al., 2015; Schwen et al., 2016). In general, blood flow modeling aims to reveal the hemodynamic features of hepatic circulation under healthy and pathological conditions (Audebert et al., 2017; Ho and Qiu, 2019), and these models may be grouped into different spatial scales. At the liver organ and hepatic vasculature level, the blood is treated as a continuum because the relative size of blood cells is much smaller than blood vessels. However, at the lobule level, this assumption does not hold because the diameter of sinusoids (23.5 μm) is similar to that of red blood cells (Wambaugh and Shah, 2010). Thus, a different modeling strategy for liver lobules is required. Considering the very complex morphological structure of liver lobules, much simplified lobular geometries, e.g., a hexagon with evenly distributed sinusoids and hepatocytes, are usually adopted (Ricken et al., 2015; Cherkaoui-Rbati et al., 2017). An even simpler lobule representation is a series of compartments arranged according to the above mentioned metabolic zones along the PT-CV axis (Fan et al., 2010; Schwen et al., 2016), as shown in Figure 1E. This zonal representation has been adopted by several pharmacokinetic (PK) studies dealing with metabolic heterogeneity (Fu et al., 2018; Franiatte et al., 2019). Putting together, simulations for the blood and drug flow, uptake, excretion, and metabolism of drugs in hepatocytes constitute a typical example of multiscale modeling, as illustrated in Figure 1.

To build an integrated multiscale platform for the hepatic blood flow and drug transport, the variety of models at different spatial and temporal scales need to be connected (Christ et al., 2017). Multiscale modeling for the liver has become an intensive research area over the past one decade, e.g., for the metabolism and clearance of acetaminophen (Sluka et al., 2016), hepatic virus B infection dynamics (Cangelosi et al., 2017), the drug-drug interaction (DDI) of midazolam (Cherkaoui-Rbati et al., 2017), to name a few research topics. Among these *in silico* simulations, virtual lobule models play a central role by linking macro- and microscale blood flow and drug uptake/metabolism models. The aim of this paper is to provide a mini-review of these works, and to provide a roadmap for multiscale biomechanical and pharmacological modeling for the liver.

## Multi-Dimensional Models for Hepatic Circulation

Different mathematical/computational techniques are used for modeling the hepatic blood flow. When subject-specific vascular information are essential, for example, in radiotherapies where drugs are administrated through a catheter at a specific vascular site (Simoncini et al., 2018), vascular geometry is included in the blood flow model in one or three dimensional (1D or 3D) partial differential equations (Ho et al., 2013b; Audebert et al., 2017). When the blood flow in vessels is considered as a steady Poiseulle flow, the flow equations can be parameterised per the vascular diameter, and length and effectively solved (Barléon et al., 2018). Fast graph or distributed network- based method can be applied to sinusoidal flow (Wambaugh and Shah, 2010), or the hepatic flow in thousands of hepatic vessels (Barléon et al., 2018).

For medical image-based flow simulations, the diameter of the smallest vessels digitized from clinical CT/MRI imaging is about 1 mm. To reach sinusoids (23.5 μm) in liver lobules, tree growing algorithms such as the constructive constrained optimisation (CCO) algorithm are used to extend hepatic vascular trees for several generations, until reaching the peripheral PTs of lobules (Schwen et al., 2015; Muller et al., 2017). For example, a CCO algorithm-generated PT tree shown in Figure 1A contains more than 8,000 vessels, ranging from the root PV (diameter ~10 mm) to lobular level portal triads (diameter ~50 μm) (Barléon et al., 2018).

When the exact vascular geometry is not required but systemic circulation features are the modeling focus, electrical analog, or 0D models are used, for example, to quantify the hepatic venous pressure gradient (Wang et al., 2017), the hepatic arterial buffer response (Ho et al., 2013a; Becker et al., 2019). 0D models have also been used to simulate blood flows in hepatectomy (Yu et al., 2020).

## Pharmacokinetics Models for The Liver

PK models aim to quantify the drug absorption, disposition, metabolism, and excretion (ADME) in the human body. They may be grouped into physiologically based pharmacokinetic (PBPK) models at the whole-body scale (Jones and Rowland-Yeo, 2013) (one such a model is shown in Figure 1D), and liver-specific models as shown in Figure 1E. In PBPK models, the liver compartment is treated as “well-stirred,” i.e., the drug concentration is evenly distributed in the organ (Jones and Rowland-Yeo, 2013). By contrast, the “parallel-tube” model assumes plug flow where drug concentration decays in an exponential fashion along the length of a sinusoid (Liu and Pang, 2006), and the “dispersion” model where a certain degree of mixing between sinusoidal blood and lateral hepatocytes occurs (Liu and Pang, 2006).

To quantify the transmembrane transport and metabolism as other determinants of hepatic drug clearances, liver-specific models include the liver tissue, sinusoids and biliary tracts in separate compartments (Liu and Pang, 2006; Meyer et al., 2017; Audebert and Vignon-Clementel, 2018), as shown in the blue colored compartments in Figure 1D. An extra layer of complexity arises when metabolic heterogeneity is considered, where these compartments are grouped in units to represent metabolic zones (Meyer et al., 2017) (Figure 1D).

## Virtual Liver Lobule Models

The dimension of liver lobule is about 1 mm, i.e., sits between the spatial scales of hepatic vessels (1–10 mm) and hepatocytes (~10 μm). Many virtual lobule models have been proposed for the sinusoidal flow, the drug perfusion and/or active transport. The research questions for modelers are therefore to implement the interface between the blood and hepatocytes, and between the hepatocytes and intra-cellular chemical species.

Concerning the morphological layout of liver lobules, a concept of “sinusoidal segment” (SS) is proposed in Sheikh-Bahaei et al. (2010), where the hepatocytes in a lobule is grouped into hundreds of SSs. Each SS is a software agent that can be used to schedule its own events. In Ohno et al. (2008), the kinetics of ammonia detoxification is incorporated into the eight compartments arranged in a series along the PT-CV axis, each has its own set of ammonia metabolism parameters. Similar strategy is used in Meyer et al. (2017), where a series of cytoplasm and bile canaliculi compartments are grouped into three units to simulate the heterogenous efflux of fluorescent tracer CFDA across three metabolic zones. In Diaz Ochoa et al. (2013), a different strategy for lobule is used, where each of the six representative sinusoids in a liver lobule transports blood from PT to CV. Moreover, metabolic zonation is prescribed by assuming the CYP3A4 (the metabolism enzyme for acetaminophen) activity is similar in Zones 1 and 2, but 1.3 times higher in Zone 3. The model thus simulates the necrosis of hepatocytes starting from Zone 3 after a bolus overdose of acetaminophen (Diaz Ochoa et al., 2013).

In the micro-dosimetry model of Wambaugh and Shah (2010), hepatocytes are arranged in several 2D plates aligned along a 3D polyhedron, and each 2D plate consists of six PTs branching toward the CV. In comparison, an anatomically accurate model is presented in Hoehme et al. (2010), where the sinusoid network and hepatocytes are constructed based on 3D confocal laser scan images. Such a lobular structure has been employed to study the liver tissue regeneration after damage by CCl_4_ (Hoehme et al., 2010), and ammonia detoxification (Schliess et al., 2014).

From a mathematical modeling's perspective, the sinusoidal flow may be modeled with a partial differential equation (PDE), where the spatial variable *x* transverses a sinusoid, and the temporal variable *t* spans a designated time. The dependant variable *c* is the concentration of a drug in the blood, while *c*_*Tissue*_ is the drug concentration in hepatocytes or liver tissue (Franiatte et al., 2019). The influx and efflux of drug molecules across the sinusoidal wall are modeled by the two terms inside the red block in the listed equation in Figure 1. When the spatial dimension is not considered but the temporal profile is critical, such as the time course of drug concentration in the liver, ordinary differential equations (ODEs) are used to quantify the drug metabolism in hepatocytes or liver tissues (Reddyhoff et al., 2015; Franiatte et al., 2019). In this way, cellular and intra-cellular dynamics are coupled. This approach has been used in a number of studies, e.g., to describe the glucose homeostasis where the metabolism kinetics of glucose, lactate, and glycogen is coupled with the finite element model of sinusoids (Ricken et al., 2015). The zonal hepatotoxicity due to overdose of acetaminophen is simulated in a similar fashion (Franiatte et al., 2019; Means and Ho, 2019).

It is worth stressing that simulation results need to be compared with *in vivo*/*in vitro* measurements and/or clinical observations. For example, blood flow simulations need to be validated by Doppler ultrasound measurements, or blood pressure measured invasively with a catheter or non-invasively with blood pressure devices. Simulated drug concentration profiles from PK models need to be compared with that measured from blood, or from cell line data (Liu and Pang, 2006). Moreover, PK models are prone to errors, as PK parameters (e.g., the volume of distribution, drug clearance parameters) are often not available but have to be estimated. Hence, parameter analysis methods, such as the Latin Hypercube sampling method (Zhang et al., 2020), the genetic algorithm (Koza, 1992), are very helpful and should be routinely practiced. In addition, errors in biomechanical models may arise from the choice of numerical schemes. For example, when solving arterial flow equations, the selection of mesh sizes and temporal steps must meet the Courant–Friedrichs–Lewy (CFL) condition to avoid numerical instabilities and errors (Du et al., 2016).

## Discussion and Conclusion

In the pharmaceutical industry, there is a tremendous competition to develop innovative therapies in a highly regulated environment (Leil and Ermakov, 2015). The research and development (R&D) costs for bringing new drugs to market is as high as ~$1.3 billion per drug (Leil and Ermakov, 2015). Liver, the major organ for drug metabolism and detoxification, has been modeled in different aspects and with different numerical approaches. In this mini-review, we have briefly introduced some recent virtual lobule models which bridge biomechanical models of blood flow and PK models in the liver. We have outlined a multiscale framework for the connections between models at different scales, as shown in Figure 1.

There are many applications for such a framework. For example, one may consider Small for Size Syndrome (SFSS) after hepatectomy or liver transplantation (Gondolesi, 2002), where the portal flow in the remnant liver increases radically, in some cases almost doubled post-surgery (Gondolesi, 2002). At the sinusoidal level, the flow rate increases accordingly, leading to an elevated shear stress and rate, which may damage sinusoidal cells (Li et al., 2010). Therefore, a biomechanical model needs to address blood flow in different scales, i.e., at the organ and sinusoidal levels. Pharmaceutical therapies, such as application of somatostatin, are used to treat the symptoms by reducing the portal blood flow (Xu et al., 2006). This is a typical scenario for combined biomechanical and pharmacological modeling. Numerical challenges arise, for example, while the pulsatile arterial flow can be characterized by the pressure and velocity waveforms for several cardiac cycles lasting for several seconds, the clearance of xenobiotic agents may require hours or even days. Tuning the parameters in such a framework allows simulating various pathological conditions, e.g., portal hypertension, steatosis in fatty liver donors, which are otherwise difficult or costly to experiment and observe in an *in vivo* or *in vitro* setup.

Future directions to apply the *in silico* framework lie on novel applications employing experimental data, such as the data from genome-wide reconstruction of the spatial zonation in the liver (Halpern et al., 2017), where the entire transcriptome of thousands of mouse liver cells have been used to infer a panel of zonated landmark genes. This kind of data have important basic research and clinical implications, and could be utilized to tweak the heterogeneity parameters (e.g., the influx and efflux parameters in the listed equation in Figure 1). The ultimate aim is to aid clinical research, and to reduce R&D costs in drug development.

In conclusion, a multiscale modeling framework has been introduced for the liver, in particular for liver lobules. We deem this research direction for hepatic circulation and pharmacokinetics is very promising in innovative drug development as well as hepatology research.

## Author Contributions

HH and EZ conceptualized the paper. HH drafted the paper. EZ reviewed the paper and provided the pharmacological context. All authors contributed to the article and approved the submitted version.

## Conflict of Interest

The authors declare that the research was conducted in the absence of any commercial or financial relationships that could be construed as a potential conflict of interest.

## References

[B1] Audebert C. Bucur P. Bekheit M. Vibert E. Vignon-Clementel I. E. Gerbeau J.-F. (2017). Kinetic scheme for arterial and venous blood flow, and application to partial hepatectomy modeling. Comput. Methods Appl. Mech. Eng. 314, 102–125. 10.1016/j.cma.2016.07.009

[B2] Audebert C. Vignon-Clementel I. E. (2018). Model and methods to assess hepatic function from indocyanine green fluorescence dynamical measurements of liver tissue. Eur. J. Pharm. Sci. 115, 304–319. 10.1016/j.ejps.2018.01.008 29339226

[B3] Barléon N. Clarke R. J. Ho H. (2018). Novel methods for segment-specific blood flow simulation for the liver. Comput. Methods Biomech. Biomed. Engin. 21, 780–783. 10.1080/10255842.2018.1520224 30398063

[B4] Becker D. Hefti M. Schuler M. Borrego L. B. Hagedorn C. Muller X.. (2019). Model assisted analysis of the hepatic arterial buffer response during *ex vivo* porcine liver perfusion. IEEE Trans. Biomed. Eng. 67, 667–678. 10.1109/TBME.2019.2919413 31150329

[B5] Cangelosi Q. Means S. A. Ho H. (2017). A multi-scale spatial model of hepatitis-B viral dynamics. PLoS ONE 12:e0188209. 10.1371/journal.pone.0188209 29216213 PMC5720747

[B6] Cherkaoui-Rbati M. H. Paine S. W. Littlewood P. Rauch C. (2017). A quantitative systems pharmacology approach, incorporating a novel liver model, for predicting pharmacokinetic drug-drug interactions. PLoS ONE 12:e0183794. 10.1371/journal.pone.0183794 28910306 PMC5598964

[B7] Christ B. Dahmen U. Herrmann K.-H. König M. Reichenbach J. R. Ricken T.. (2017). Computational modeling in liver surgery. Front. Physiol. 8:906. 10.3389/fphys.2017.00906 29249974 PMC5715340

[B8] Diaz Ochoa J. G. Bucher J. Pery A. R. R. Zaldivar Comenges J. M. Niklas J. Mauch K. (2013). A multi-scale modeling framework for individualized, spatiotemporal prediction of drug effects and toxicological risk. Front. Pharmacol. 3:204. 10.3389/fphar.2012.00204 23346056 PMC3551257

[B9] Du T. Hu D. Cai D. (2016). A fast algorithm for the simulation of arterial pulse waves. J. Comput. Phys. 314, 450–464. 10.1016/j.jcp.2016.03.036

[B10] Fan J. Chen S. Chow E. C. Y. Pang K. S. (2010). PBPK modeling of intestinal and liver enzymes and transporters in drug absorption and sequential metabolism. Curr. Drug Metab. 11, 743–761. 10.2174/138920010794328931 21189137

[B11] Franiatte S. Clarke R. Ho H. (2019). A computational model for hepatotoxicity by coupling drug transport and acetaminophen metabolism equations. Int. J. Numer. Method. Biomed. Eng. 35:e3234. 10.1002/cnm.3234 31254976

[B12] Fu X. Sluka J. P. Clendenon S. G. Dunn K. W. Wang Z. Klaunig J. E.. (2018). Modeling of xenobiotic transport and metabolism in virtual hepatic lobule models. PLoS ONE 13:e0198060. 10.1371/journal.pone.0198060 30212461 PMC6136710

[B13] Gondolesi G. (2002). Venous hemodynamics in living donor right lobe liver transplantation. Liver Transplant. 8, 809–813. 10.1053/jlts.2002.33690 12200783

[B14] Halpern K. B. Shenhav R. Matcovitch-Natan O. Tóth B. Lemze D. Golan M.. (2017). Single-cell spatial reconstruction reveals global division of labour in the mammalian liver. Nature 542, 352–356. 10.1038/nature21065 28166538 PMC5321580

[B15] Ho H. Qiu C. (2019). Hemodynamic aspects of the Budd–Chiari syndrome of the liver: a computational model study. Med. Eng. Phys. 69, 134–139. 10.1016/j.medengphy.2019.04.010 31078370

[B16] Ho H. Sorrell K. Bartlett A. Hunter P. (2013a). Modeling the hepatic arterial buffer response in the liver. Med. Eng. Phys. 35, 1053–1058. 10.1016/j.medengphy.2012.10.008 23157977

[B17] Ho H. Sorrell K. Peng L. Yang Z. Holden A. Hunter P. (2013b). Hemodynamic analysis for transjugular intrahepatic portosystemic shunt (TIPS) in the liver based on a CT-Image. IEEE Trans. Med. Imaging 32, 92–98. 10.1109/TMI.2012.2219882 23014713

[B18] Hoehme S. Brulport M. Bauer A. Bedawy E. Schormann W. Hermes M.. (2010). Prediction and validation of cell alignment along microvessels as order principle to restore tissue architecture in liver regeneration. Proc. Natl. Acad. Sci. U.S.A. 107, 10371–10376. 10.1073/pnas.0909374107 20484673 PMC2890786

[B19] Jones H. M. Rowland-Yeo K. (2013). Basic concepts in physiologically based pharmacokinetic modeling in drug discovery and development. CPT Pharm. Syst. Pharmacol. 2:e63. 10.1038/psp.2013.41 23945604 PMC3828005

[B20] Jungermann K. (1995). Zonation of metabolism and gene expression in liver. Histochem. Cell Biol. 103, 81–91. 10.1007/BF01454004 7634156

[B21] Kleiner D. E. Brunt E. M. (2012). Nonalcoholic fatty liver disease: pathologic patterns and biopsy evaluation in clinical research. Semin. Liver Dis. 32, 003–013. 10.1055/s-0032-1306421 22418883

[B22] Koza J. R. (1992). Genetic Programming : On the Programming of Computers by Means of Natural Selection. Cambridge, MA: MIT Press.

[B23] Leil T. A. Ermakov S. (2015). Editorial: The emerging discipline of quantitative systems pharmacology. Front. Pharmacol. 6:129. 10.3389/fphar.2015.00129 26175687 PMC4485322

[B24] Li J. Liang L. Ma T. Yu X. Chen W. Xu G.. (2010). Sinusoidal microcirculatory changes after small-for-size liver transplantation in rats. Transplant. Int. 23, 924–933. 10.1111/j.1432-2277.2010.01058.x 20210931

[B25] Liu L. Pang K. S. (2006). An integrated approach to model hepatic drug clearance. Eur. J. Pharma. Sci. 29, 215–230. 10.1016/j.ejps.2006.05.007 16806855

[B26] Means S. A. Ho H. (2019). A spatial-temporal model for zonal hepatotoxicity of acetaminophen. Drug Metab. Pharmacokinet. 34, 71–77. 10.1016/j.dmpk.2018.09.266 30377056

[B27] Meyer K. Ostrenko O. Bourantas G. Morales-Navarrete H. Porat-Shliom N. Segovia-Miranda F.. (2017). A predictive 3D multi-scale model of biliary fluid dynamics in the liver lobule. Cell Syst. 4, 277–290.e9. 10.1016/j.cels.2017.02.008 28330614 PMC8063490

[B28] Muller A. Clarke R. Ho H. (2017). Fast blood-flow simulation for large arterial trees containing thousands of vessels. Comput. Methods Biomech. Biomed. Engin. 20, 160–170. 10.1080/10255842.2016.1207170 27376402

[B29] Ohno H. Naito Y. Nakajima H. Tomita M. (2008). Construction of a biological tissue model based on a single-cell model: a computer simulation of metabolic heterogeneity in the liver lobule. Artif. Life 14, 3–28. 10.1162/artl.2008.14.1.3 18171128

[B30] Reddyhoff D. Ward J. Williams D. Regan S. Webb S. (2015). Timescale analysis of a mathematical model of acetaminophen metabolism and toxicity. J. Theor. Biol. 386, 132–146. 10.1016/j.jtbi.2015.08.021 26348886

[B31] Ricken T. Werner D. Holzhütter H. G. König M. Dahmen U. Dirsch O. (2015). Modeling function–perfusion behavior in liver lobules including tissue, blood, glucose, lactate and glycogen by use of a coupled two-scale PDE–ODE approach. Biomech. Model. Mechanobiol. 14, 515–536. 10.1007/s10237-014-0619-z 25236798

[B32] Schliess F. Hoehme S. Henkel S. G. Ghallab A. Driesch D. Böttger J.. (2014). Integrated metabolic spatial-temporal model for the prediction of ammonia detoxification during liver damage and regeneration. Hepatology 60, 2040–2051. 10.1002/hep.27136 24677161

[B33] Schwen L. O. Kuepfer L. Preusser T. (2016). Modeling approaches for hepatic spatial heterogeneity in pharmacokinetic simulations. Drug Discov. Today 22, 35–43. 10.1016/j.ddmod.2017.09.00226222615

[B34] Schwen L. O. Wei W. Gremse F. Ehling J. Wang L. Dahmen U.. (2015). Algorithmically generated rodent hepatic vascular trees in arbitrary detail. J. Theor. Biol. 365, 289–300. 10.1016/j.jtbi.2014.10.026 25451523

[B35] Sheikh-Bahaei S. Maher J. J. Anthony Hunt C. (2010). Computational experiments reveal plausible mechanisms for changing patterns of hepatic zonation of xenobiotic clearance and hepatotoxicity. J. Theor. Biol. 265, 718–733. 10.1016/j.jtbi.2010.06.011 20541559 PMC4126406

[B36] Simoncini C. Jurczuk K. Reska D. Esneault S. Nunes J.-C. Bellanger J.-J.. (2018). Towards a patient-specific hepatic arterial modeling for microspheres distribution optimization in SIRT protocol. Med. Biol. Eng. Comput. 56, 515–529. 10.1007/s11517-017-1703-1 28825200

[B37] Sluka J. P. Fu X. Swat M. Belmonte J. M. Cosmanescu A. Clendenon S. G.. (2016). A Liver-Centric Multiscale Modeling Framework for Xenobiotics. PLoS ONE 11:e0162428. 10.1371/journal.pone.0162428 27636091 PMC5026379

[B38] Wambaugh J. Shah I. (2010). Simulating Microdosimetry in a Virtual Hepatic Lobule. PLoS Comput. Biol. 6:e1000756. 10.1371/journal.pcbi.1000756 20421935 PMC2858695

[B39] Wang T. Liang F. Zhou Z. Shi L. (2017). A computational model of the hepatic circulation applied to analyze the sensitivity of hepatic venous pressure gradient (HVPG) in liver cirrhosis. J. Biomech. 65, 23–31. 10.1016/j.jbiomech.2017.09.023 29042056

[B40] Xu X. Man K. Zheng S. S. Liang T. B. Lee T. K. Ng K. T.. (2006). Attenuation of acute phase shear stress by somatostatin improves small-for-size liver graft survival. Liver Transplant. 12, 621–627. 10.1002/lt.20630 16555322

[B41] Yu H. B. Bartlett A. Hunter P. Ho H. (2020). Computational simulations for the hepatic arterial buffer response after liver graft transplantation from an adult to a child. Med. Eng. Phys. 75, 49–52. 10.1016/j.medengphy.2019.11.003 31734014

[B42] Zhang S. Zhang E. Ho H. (2020). Extrapolation for a pharmacokinetic model for acetaminophen from adults to neonates: A Latin Hypercube Sampling analysis. Drug Metab. Pharmacokinet. 35, 329–333. 10.1016/j.dmpk.2020.03.004 32307228

